# Effects of buprenorphine on pain perception in healthy adults: a meta-narrative systematic review

**DOI:** 10.1097/PR9.0000000000001294

**Published:** 2025-05-20

**Authors:** W. Michael Hooten, Danielle Zheng, Nicholas C. Canzanello, Rajat N. Moman, Larry Prokop, Nathan D. Eberhart, Barlas Benkli, Salman Hirani

**Affiliations:** a Division of Pain Medicine, Department of Anesthesiology and Perioperative Medicine, Mayo Clinic, Rochester, MN, USA; b Anesthesiology Systematic Review Group, Department of Anesthesiology and Perioperative Medicine, Mayo Clinic, Rochester, MN, USA; c Department of Anesthesiology, University of Pittsburgh Medical Center, Pittsburgh, PA, USA; d Department of Physical Medicine and Rehabilitation, Mayo Clinic, Rochester, MN, USA; e Pain and Spine Centers of Florida, Lady Lake, FL, USA; f Mayo Clinic Libraries, Rochester, MN, USA; g Department of Neurology, University of Florida College of Medicine, Gainesville, FL, USA; h Division of Pain Medicine, Department of Anesthesiology and Perioperative Medicine, Oregon Health & Science University, Portland, OR, USA

**Keywords:** Systematic review, Meta-narrative, Buprenorphine, Heat pain perception, Cold pain perception, Conditioned pain modulation, Nociceptive flexion reflex, Intravenous, Transdermal, Sublingual, Healthy subjects

## Abstract

The availability of knowledge about the effects of buprenorphine on pain perception in healthy adults could help investigators select experimental tests with evidence of responsiveness to buprenorphine in nondiseased states. The purpose of this meta-narrative systematic review is to summarize the effects of buprenorphine on pain perception in healthy adults. Comprehensive database searches were conducted from inception dates through July 16, 2024. Inclusion criteria included all study designs involving healthy adults receiving buprenorphine and tests of pain perception. Risk of bias was assessed using a tool developed for randomized controlled trials (RCTs). A meta-narrative approach was used due to high levels of clinical heterogeneity. A total of 10 RCTs were included, and healthy adults comprised the comparator groups in all trials. Three RCTs had a low risk of bias, 6 had some concerns, and 1 had a high risk of bias. Six RCTs used intravenous buprenorphine, 3 used transdermal, and 1 study used intravenous and sublingual buprenorphine. The effects of buprenorphine on pain perception are influenced by the route of administration, dose, and pain stimulus modality. Considerable differences exist in the effects of intravenous and transdermal buprenorphine, but only 1 study used sublingual buprenorphine, which limited comparison with the other drug formulations. Based on these findings, recommendations for clinical trials are proposed. These recommendations should be cautiously interpreted due to the clinical heterogeneity, but they provide a framework for advancing the use of experimental pain tests in trials of buprenorphine involving comparator groups of healthy adults.

## Abstract 2

Supplemental Digital Content is Available in the Text.

Considerable differences exist in the effects of buprenorphine on pain perception in healthy adults related to the route of administration, dose, and stimulus modality.

## 1. Introduction

Buprenorphine is a semisynthetic partial opioid agonist with a distinct opioid receptor binding profile. The drug has partial agonist affinity for the mu-opioid receptor, low agonist binding affinity for the opioid receptor-like 1 receptor, and high antagonist binding affinity for the delta- and kappa-opioid receptors.^13,19,22^ Buprenorphine has analgesic properties,^20^ but compared with full opioid agonists, respiratory depression,^5^ euphoria,^23^ and abuse^14^ are less likely to occur. These favorable pharmacological effects are also attributed, in part, to greater binding of spinal opioid receptors vs receptors localized in the brain.^26^ As a result of the unique binding profile, buprenorphine is widely used for maintenance-assisted therapy in people with opioid use disorder^30^ and the drug is increasingly used for chronic pain.^1,29^

Despite the widespread clinical use of buprenorphine, the effects of the drug on experimental pain perception in healthy adults have not been systematically reviewed. The availability of this knowledge could help investigators select experimental tests that have evidence of responsiveness to buprenorphine in nondiseased states. This is important because pain perception could be influenced by the route of buprenorphine administration (ie, intravenous, sublingual, or transdermal) and total administered dose. Buprenorphine may also have differential effects on experimentally induced thermal, electrical, mechanical, or pressure pain stimuli. Finally, information about the effects of buprenorphine on the nociceptive flexion reflex and conditioned pain modulation in healthy adults could support decision-making about the incorporation of these experimental tests in future clinical trials. Thus, the purpose of this meta-narrative systematic review is to describe the effects of buprenorphine on pain perception in healthy adults.

## 2. Materials and methods

### 2.1. Study protocol

Preferred Reporting Items for Systematic Reviews and Meta-Analyses (PRISMA) were followed.^15^ An a priori protocol was followed. The trial was registered in the International Prospective Register of Systematic Reviews (PROSPERO) database.^32^ The focus of the current systematic review was limited to the effects of buprenorphine on experimental pain in healthy adults.

### 2.2. Search strategy

A comprehensive search of databases from the date of inception to July 16, 2024 (date search was executed) was conducted. No language restrictions were imposed. The databases included: (1) APA PsycInfo, (2) EBM Reviews—Cochrane Central Register of Controlled Trials June 2024, (3) EBM Reviews—Cochrane Database of Systematic Reviews 2005 to July 10, 2024, (4) Embase 1974 to 2024 July 15, (5) Ovid MEDLINE and Epub Ahead of Print, In-Process, In-Data-Review & Other Non-Indexed Citations, Daily and Versions 1946 to July 15, 2024, and (6) Scopus. The search strategy was designed and executed by an experienced librarian with input from the principal investigator. Controlled vocabulary supplemented with keywords was used. The search strategy listing all search terms and how the search terms were combined is available in a supplemental file (Supplemental Material, http://links.lww.com/PR9/A317).

### 2.3. Study selection process

Study inclusion criteria included (1) randomized-, crossover-, and parallel-designed clinical trials, (2) prospective and retrospective cohort studies, (3) cross sectional studies, (4) age 18 years or older, (5) all publication years, (6) any publication language, (7) studies involving all modalities of pain perception including thermal, mechanical, electrical, conditioned pain modulation, and primary and secondary hyperalgesia, and (8) studies involving all commercially available formulations of buprenorphine. Exclusion criteria included (1) animal studies and (2) studies combining buprenorphine with other drugs, including placebo.

In the first phase, 2 independent pairs of reviewers screened all titles and abstracts identified by the search strategy. In the second phase, all full-text articles were screened for inclusion and exclusion criteria and the reason for exclusion of each full text was noted.

### 2.4. Data extraction

Data were extracted by 4 independent reviewers using a templated electronic database.

Based on the a priori protocol, abstracted data included study design, number of subjects, demographics (mean age, percent male sex), buprenorphine dose and route of administration, duration of buprenorphine administration, and quantitative sensory testing methodology. At least 2 attempts were made to contact investigators for incompletely reported outcomes. In the Andresen et al.^2^ randomized controlled trial (RCT), a discrepancy occurred in the abstraction of data pertaining to the use of ultraviolet B light (W.M.H., N.D.E.). The discrepancy was resolved by consensus following the review of the published article (see Results section, Primary and secondary hyperalgesia subsection).

### 2.5. Risk of bias assessment

The risk of bias in the included studies was assessed by 2 independent reviewers using the Risk of Bias 2.0 for RCTs tool.^25^ A summary risk of bias was reported for each trial included in the systematic review. Reviewer discrepancy was resolved by consensus or by a third reviewer.

### 2.6. Evidence synthesis

Owing to the heterogeneity in study characteristics and the small number of studies, a meta-analysis was not performed; alternatively, the results are presented using a meta-narrative approach. A meta-narrative approach is appropriate when the subject matter of a systematic review has been researched using divergent methods.^7,31^ In addition, this approach is particularly useful when key clinical factors and definitions vary between studies.^7,31^

## 3. Results

### 3.1. Characteristics of included studies

**Figure 1.**
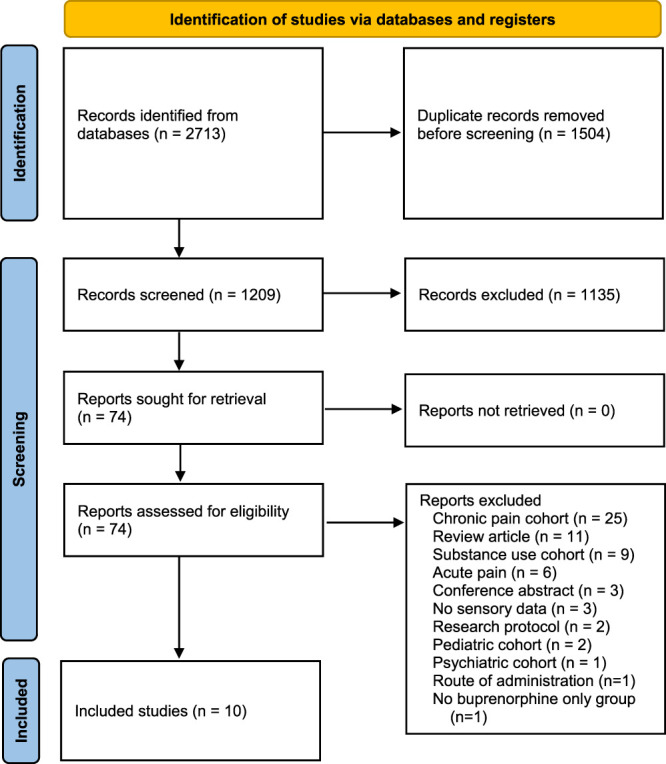
PRISMA 2020 flow diagram for systematic reviews, which included searches of databases and registers only. *From:* Page MJ, McKenzie JE, Bossuyt PM, Boutron I, Hoffmann TC, Mulrow CD, et al. The PRISMA 2020 statement: an updated guideline for reporting systematic reviews. BMJ 2021;372:n71. Adaptations are themselves works protected by copyright. So, to publish this adaptation, authorization must be obtained both from the owner of the copyright in the original work and from the owner of the copyright in the translation or adaptation.

Figure 1 depicts a flow diagram of the study selection process. A total of 10 studies met the inclusion and exclusion criteria.^2,3,5,6,8–10,12,21,27^ All 10 studies were RCTs. The publications by Andresen et al.^2^ and Arendt-Nielsen et al.^3^ reported different data points from the same RCT. Modes of buprenorphine administration included intravenous,^5,6,8,10,12,21,27^ transdermal,^2,3,9^ and sublingual.^10^ Sensory modalities used to assess pain perception included heat pain,^2,3,6,9^ coolness,^21^ cold pain,^6^ cold pressor pain,^2,3,6,8,9,12,21^ ischemic pain, mechanical pain,^2,3,10,21^ and electrical pain (Table 1).^2,3,5,9,10,27^

**Table 1:** Study characteristics.

Study	Design	Population	Number subjects	Mean age (SD)	Sex (% men)	Intervention	Stimulus modality	Body region of testing	Study time range	Comments
Andresen et al.,^2^ 2011	Randomized, double-blind, placebo-controlled, crossover	Healthy subjects	20	23.1 y (3.8)	100%	Bup 20 μg/h TD for 144 h	Mechanical pressure pain tolerance (handheld algometer) Heat pain tolerance (Medoc device) Electrical pain threshold	Right tibia 15 cm below patella Right volar forearm Left volar forearm over median nerve	Three 10-d sessions (7 d of treatment, 3 d follow-up); tested at time 0, 24, 48, 72, 144 h	Same clinical trial and cohort as Arendt-Nielsen 2012
Arendt-Nielsen et al.,^3^ 2012	Randomized, double-blind, placebo-controlled, crossover	Healthy subjects	20	23.1 y (3.8)	100%	Bup 20 μg/h TD for 144 h	Cold pressor tolerance (water 3°C) Mechanical pressure pain tolerance (handheld algometer)	Right hand Lateral left arm	Three 10-d sessions (7 d of treatment, 3 d follow-up); tested at time 0, 24, 48, 72, 144 h	Same clinical trial and cohort as Andresen 2011
Dahan et al.,^5^ 2006	Randomized, double-blind, placebo-controlled	Healthy patients	20	22–35 y	50%	Bup 0.2 mg/70 kg IV or bup 0.4 mg/70 kg IV as single dose over 90 s	Electrical pain tolerance (TC)	Tibia (left)	One 8-h session (tested at t = 0, 5, 10, 45, 80, 110, 130, 165, 210, 270, 330, 390, 450, 490 min)	
Escher et al.,^6^ 2007	Randomized, double-blind, placebo-controlled, crossover	Healthy subjects	12	26 y (3.5)	100%	Bup 2 μg/kg IV over 1 min	Heat pain threshold (Medoc device) Cold pain threshold (Medoc device) Cold pressor pain tolerance (water 0°C) Electrical pain threshold and NFR threshold	Thermode on ventral forearm Thermode on ventral forearm Hand (laterality not specified) Sural nerve in retromalleolar track and tendon biceps femoris	Three 8-h sessions; tested at time 0, 0.5, 1, 1.5, 2, 3, 4, 6, 8 h	
Hay et al.,^8^ 2011	Randomized, double-blind, crossover	Healthy patients	10	23 y (1.3)	50%	Bup 0.5 μg/kg IV over 30 min	Cold pressor tolerance (water 0.5° to 1.5°C)	Nondominant forearm	Five 1-d sessions (tested at time 0, 30, 90, 150, 210, 290 min)	Naltrexone data excluded
Koltzenburg et al.,^9^ 2006	Randomized, double-blind, placebo-controlled, crossover	Healthy subjects	20	32.4 y (range 19–42)	75%	Bup 35 μg/h TD for 72 h	Heat pain threshold and tolerance (Medoc device) Cold pressor AUC pain intensity (water 1–2°C) Electrical pain tolerance (TC)	Forearm (left) Hand (nondominant) Lateral upper arm (nondominant)	Four 72-h sessions; tested at time 0, 24, 72 h	
Koppert et al.,^10^ 2005	Randomized, double-blind, placebo-controlled, crossover	Healthy subjects	15	28.5 y (5)	60%	Bup 1.5 μg/kg IV over 5 min Bup 0.2 mg sublingual	Continuous electrical stimulus titrated to NRS pain score of 6 Mechanical pinprick; 450 mN von Frey filament	Central volar region of forearm Central volar region of forearm	Four 150-min sessions at intervals of 2 wk	
La Vincente et al.,^12^ 2008	Randomized, double-blind, crossover	Healthy subjects	13	27.5 y (7.8)	46%	Bup 0.5 μg/kg IV over 30 min	Cold pressor tolerance (water 0.5° to 1.5°C)	Nondominant forearm	Four 1-d sessions (tested at time 0, 30, 90, 150, 210, 270, 330, 390 min)	Excluded naloxone data
Ravn et al.,^21^ 2013	Randomized, double-blind, placebo-controlled, crossover	Healthy subjects	28	25.1 y (3.8)	54%	Bup 0.3 mg or bup 0.6 mg IV as single dose over 210 min	Cold pressor threshold and tolerance (water 0.3°C) Cool detection threshold Mechanical pain tolerance (pinprick algometer)	Nondominant hand Dominant index finger	Five 8-h sessions; tested after completion of infusion	Excluded heat-induced injury pain test results
Tröster et al.,^27^ 2012	Randomized, double-blind, placebo-controlled, crossover	Healthy subjects	15	27 y (4) Range 22–35 y	100%	Bup 1.5 μg/kg IV over 10 min	Continuous electrical stimulus titrated to NRS pain score of 6 Mechanical pinprick; 450 mN von Frey filament	Central volar region of forearm Central volar region of forearm	Four 150-min sessions at intervals of 2 wk	

Bup, buprenorphine; IV, intravenous; NFR, nociceptive flexion reflex; NRS, numeric rating scale; TD, transdermal.

### 3.2. Risk of bias

Table 2 contains the summary results of the risk of bias evaluation. Three RCTs had a low risk of bias, 6 had some concerns about bias, and 1 RCT had a high risk of bias. The Ravn et al.^21^ RCT had a high risk of bias because half of the participants were excluded from analysis.

**Table 2:** Risk of bias 2.0 assessment.

Study	Bias from randomization process	Bias due to deviations from intended interventions	Bias due to missing outcome data	Bias in measurement of the outcome	Bias in selection of the reported result	Overall risk of bias judgment
Andresen et al.,^2^ 2011	Some concerns	Low	Low	Low	Low	Some concerns
Arendt-Nielsen et al.,^3^ 2012	Some concerns	Low	Low	Low	Low	Some concerns
Dahan et al.,^5^ 2006	Some concerns	Some concerns	Some concerns	Low	Low	Some concerns
Escher et al.,^6^ 2007	Low	Low	Low	Low	Some concerns	Some concerns
Hay et al.,^8^ 2011	Low	Low	Low	Low	Low	Low
Koltzenburg et al.,^9^ 2006	Low	Low	Some concerns	Low	Some concerns	Some concerns
Koppert et al.,^10^ 2005	Low	Low	Low	Low	Low	Low
La Vincente et al.,^12^ 2008	Low	Low	Some concerns	Low	Low	Some concerns
Ravn et al.,^21^ 2013	Low	Low	High risk	Some concerns	Some concerns	High risk
Tröster et al.,^27^ 2012	Low	Low	Low	Low	Low	Low

### 3.3. Pain perception in healthy adults

#### 3.3.1. Heat pain perception

Three studies measured heat pain perception in healthy subjects.^2,6,9^ One RCT used intravenous buprenorphine,^6^ and 2 trials used transdermal buprenorphine.^2,9^ Heat pain stimuli were delivered by contact thermodes in all studies with a device using the method of limits.^2,6,9^

##### 3.3.1.1. Heat pain threshold

###### 3.3.1.1.1. Intravenous buprenorphine

In the Escher et al.^6^ RCT, intravenous buprenorphine 2 μg/kg administered over 1 minute had no significant effect on heat pain thresholds, but numerical values were not reported.

###### 3.3.1.1.2. Transdermal buprenorphine

Following the application of transdermal buprenorphine 35 μg/h in the Koltzenburg et al.^9^ RCT, mean heat pain tolerance increased by 0.83°C (SD 2.38, *P* < 0.05) and 1.59°C (SD 2.35, *P* < 0.05) above the mean baseline value of 42.88°C (SD 3.55) at 24 hours and 72 hours, respectively.

##### 3.3.1.2. Heat pain tolerance

###### 3.3.1.2.1. Transdermal buprenorphine

Heat pain tolerance was assessed in 2 RCTs.^2,9^ Following the application of transdermal buprenorphine 20 μg/h in the Andresen et al.^2^ RCT, mean heat pain tolerance increased by 0.5°C, 2.1°C, and 1.2°C above baseline values at 24 hours, 48 hours, and 72 hours, respectively. The values of change in heat pain tolerance were presented graphically, and exact numerical values and standard errors of the mean were not reported. A 2-way ANOVA analysis demonstrated greater changes in heat pain tolerance within 72 hours compared with placebo (*P* < 0.001). At 144 hours following the application of transdermal buprenorphine, the change in heat pain tolerance was significantly greater (1.2°C) compared with placebo (*P* = 0.03).

In the Koltzenburg et al.^9^ RCT, no values for heat pain tolerance were reported.

#### 3.3.2. Cold pain perception

Four RCTs measured cold pressor pain in subjects receiving intravenous buprenorphine,^6,8,12,21^ 2 RCTs measured cold pressor pain in subjects receiving transdermal buprenorphine,^3,9^ and 1 RCT assessed cool detection thresholds using intravenous buprenorphine.^21^

##### 3.3.2.1. Cold pressor pain threshold

###### 3.3.2.1.1. Intravenous buprenorphine

In the Ravn et al.^21^ RCT, the nondominant hand was immersed 1 cm to 2 cm above the wrist in a 0.3°C to 0.5°C water bath, and the time to pain threshold was recorded. Compared with placebo, significant elevations in cold pressor pain thresholds were observed 75 minutes after initiation of an intravenous infusion of buprenorphine 0.3 mg (*P* < 0.005) and 0.6 mg (*P* < 0.001). The total infusion time of each buprenorphine dose was 210 minutes. The means and standard deviations for values of cold pressor pain threshold were presented graphically, but exact values were not reported.

##### 3.3.2.2. Cold pressor pain tolerance

###### 3.3.2.2.1. Intravenous buprenorphine

Cold pressor pain tolerance was reported in 4 studies.^6,8,12,21^ In the Hay et al.^8^ RCT, buprenorphine 0.5 μg/kg was administered intravenously over a 30-minute period. Cold pressor pain tolerance was then assessed 30 minutes postinfusion and hourly thereafter for a four-hour period. The cold pressor test was performed by immersing the nondominant forearm in a 34.5°C to 35.5°C water bath for 2 minutes, after which the arm was transferred to a 0.5°C to 1.5°C water bath. A blood pressure cuff was inflated to a pressure of 20 mm Hg below diastolic blood pressure 15 seconds before immersion of the forearm in the cold water bath. Cold pressor pain tolerance at baseline was 45.7 seconds (SD 27.2), but no significant changes in cold pain tolerance were observed at any postinfusion assessment timepoint.

In the La Vincente et al.^12^ RCT, the cold pressor pain methodology and buprenorphine dosing regimen were identical to the methods and dosing scheme used in the Hay et al.^8^ RCT. However, the time period of assessment following the completion of the buprenorphine infusion was 6 hours in the La Vincente et al.^12^ RCT vs 4 hours in the Hay et al.^8^ RCT. Cold pressor pain tolerance at baseline was 49.2 seconds (SD 28.4), but no significant changes in cold pressor pain tolerance were observed at any postinfusion assessment timepoint.

In the Escher et al.^6^ RCT, buprenorphine 2 μg/kg was administered intravenously as a single 1 mL bolus over 1 minute, and cold pressor pain tolerance was assessed at 0.5, 1, 1.5, 2, 3, 4, 6, and 8 hours afterwards. Cold pressor pain tolerance was performed by immersing the subject's hand (hand dominance not specified) in an ice water bath at 0°C (±0.1°C). The peak effect of buprenorphine on cold pressor pain tolerance occurred 30 minutes after the bolus infusion was administered. The mean time to cold pressor tolerance was 69 seconds (SEM 10), which was 63.8% (SEM 14.4) above the mean baseline value (*P* = 0.003).

In the Ravn et al.^21^ RCT, cold pressor pain tolerance was significantly elevated compared with placebo 75 minutes after administration of an intravenous infusion of buprenorphine 0.3 mg (*P* < 0.0001) and 0.6 mg (*P* < 0.0001). The total infusion time of each buprenorphine dose was 210 minutes. The means and standard deviations for values of cold pressor pain threshold were presented graphically, but exact values were not reported.

##### 3.3.2.3. Other measures of cold pressor pain

###### 3.3.2.3.1. Transdermal buprenorphine

In the Arendt-Nielsen et al.^3^ RCT, measures of cold pressor pain were assessed at baseline and at 24, 48, and 72 hours after administration of transdermal buprenorphine 20 μg/h. Cold pressor pain was assessed by immersing the right hand in a cold water bath at 3.0°C (SEM 0.3) for 2 minutes. Pain intensity was assessed using an electronic visual analogue scale (VAS), and peak pain intensity, time to peak pain intensity, and area under the VAS pain score curve were recorded. Before buprenorphine administration, the mean peak pain intensity was 8.9 (SEM 0.1), the mean time to peak pain intensity was 65 seconds (SEM 2.0), and the area under the pain intensity curve was 850 (SEM 11). Transdermal buprenorphine was associated with a nonsignificant reduction (*P* = 0.06) in the time to peak pain intensity, but no significant increase in peak pain intensity or area under the pain intensity curve was observed.

In the Koltzenburg et al.^9^ RCT, measures of cold pressor pain were assessed at baseline and at 24 and 72 hours after administration of transdermal buprenorphine 35 μg/h. Cold pressor pain was assessed by immersion of the nondominant hand in an ice-saturated bath that ranged from 1.89°C (SD 0.39) to 2.08°C (SD 0.16). Pain intensity was assessed using an electronic VAS, and peak pain intensity, area under the VAS pain score curve, and mean pain intensity were recorded. At baseline, the area under the pain intensity curve was 5000 (estimated value presented graphically). Transdermal buprenorphine was associated with a 43% reduction (*P* < 0.001) in the area under the pain intensity curve at the 24-hour assessment timepoint, and the significance of the effect was maintained at 72 hours (numerical values and statistics not reported). The mean baseline peak pain intensity was 65.16 (SD 38.49), and transdermal buprenorphine was associated with a 19.59% (SD 22.05, *P* < 0.01) and 13.36% (SD 22.20, *P* < 0.05) reduction in peak pain intensity at the 24 and 72 hours assessment timepoints, respectively. The baseline mean pain intensity was 33.51 (SD 24.62), and transdermal buprenorphine was associated with a 12.23% (SD 15.74, *P* < 0.05) and 10.69% (SD 15.18, *P* < 0.05) reduction in mean pain intensity at the 24- and 72-hour assessment timepoints, respectively.

##### 3.3.2.4. Cool detection threshold

###### 3.3.2.4.1. Intravenous buprenorphine

In the Ravn et al.^21^ RCT, cool detection thresholds were measured using a device (MSA Thermotest) based on the method of limits. The anatomical site of testing was the upper medial aspect of the nondominant lower leg. Thermal neutrality was defined as 32°C, and the rate of temperature decline was 2°C per second. Subjects were instructed to indicate when the first perceived change in temperature was detected. Cool detection thresholds were assessed at baseline, upon completion of the 210-minute intravenous infusions of buprenorphine 0.3 mg and 0.6 mg, and at 2 and 3 hours following the completion of each buprenorphine infusion. Thus, for each buprenorphine dose, cool detection thresholds were assessed at 3 postinfusion timepoints, and the mean threshold of these 3 assessments was used as the comparator values in the statistical analyses. Compared with baseline and placebo values, both buprenorphine doses were associated with significant (*P* < 0.0001) increases in cool detection thresholds. Data for cool detection thresholds were presented graphically, and exact mean values and standard error of the means were not reported.

#### 3.3.3. Mechanical pain perception

Three RCTs assessed mechanical pain perception in healthy adults following the administration of a single buprenorphine dose.^2,3,21^

##### 3.3.3.1. Mechanical pain threshold

###### 3.3.3.1.1. Intravenous buprenorphine

In the Ravn et al.^21^ RCT, mechanical pain threshold was assessed using a pinprick algometer on the dominant index finger. Subjects were instructed to indicate the threshold of pain using an electronic VAS without indicator markings. The results of pinprick algometry were not reported.

##### 3.3.3.2. Mechanical pain tolerance

###### 3.3.3.2.1. Transdermal buprenorphine

In the Andresen et al.^2^ RCT, pressure pain tolerance was assessed using a handheld algometer with a probe size of 2 mm. Force was applied at a rate of 30 kPas^−1^ to the right tibia 15 cm below the patella. A 2-way ANOVA analysis demonstrated greater changes in pressure pain tolerance within 72 hours in the transdermal buprenorphine 20 μg/h group compared with placebo (*P* = 0.007). At 144 hours, following the application of transdermal buprenorphine, the change in pressure pain tolerance was significantly greater compared with placebo (*P* = 0.05).

###### 3.3.3.2.2. Intravenous buprenorphine

In the Ravn et al.^21^ RCT, pressure pain tolerance was assessed using an electronic algometer with a 1-cm^2^ tip applied to the dorsum of the distal phalanx of the dominant index finger. Pressure pain tolerance was assessed before and immediately after completion of the cold pressor test. Testing was performed 75 minutes following the administration of the buprenorphine 0.3 mg and 0.6 mg infusions. Pressure pain tolerance was significantly lower in the placebo group before (*P* < 0.005) and immediately after (*P* < 0.005) completion of the cold pressor test compared with the buprenorphine 0.6 mg infusion group but not the 0.3 mg infusion group. In addition, the difference between the 2 measures of pressure pain tolerance was significantly smaller in the placebo group compared with the buprenorphine 0.6 mg infusion group (*P* = 0.003).

#### 3.3.4. Electrical pain perception

Three RCTs measured pain perception in response to electrical stimulation in healthy controls after a single dose of intravenous buprenorphine^5^ or administration of transdermal buprenorphine.^2,9^

##### 3.3.4.1. Electrical pain threshold

###### 3.3.4.1.1. Transdermal buprenorphine

In the Andersen et al.^2^ RCT, electrical pain threshold was assessed at baseline and then at 24, 48, 72, and 144 hours after application of buprenorphine 25 μg/h. Two different stimuli were administered: (1) single train of 5 pulses at 200 Hz, and (2) 5 stimuli each consisting of a train of 5 pulses at 2 Hz. Each of the 2 stimuli was administered 3 times, and the mean value was multiplied by 1, 1.4, 1.6, and 1.8. All subjects were then stimulated at these different intensity levels in random order. The mean baseline pain threshold for the single and repeated stimuli was 5.7 mA (SEM 1.1) and 4.4 mA (SEM 0.8), respectively. No significant differences in pain threshold were observed between the buprenorphine and placebo groups at baseline or at any of the other 4 assessment time points.

###### 3.3.4.1.2. Intravenous buprenorphine

In the Escher et al.^6^ RCT, electrical pain threshold was assessed by applying singular rectangular impulses (0.5 ms) with a 6- to 10-second interstimulus interval by a constant current stimulator. The electrical stimuli were applied to the sural nerve in the retromalleolar area following the administration of intravenous buprenorphine 2 μg/kg. Electrical pain threshold was predefined as a 4.5 pain rating on the NRS (0 = not pain, 10 = worse possible pain). The mean baseline electrical pain threshold was 45.5 mÅ (SD 22.3), and pain thresholds were significantly elevated for >4 hours. The maximum increase in pain threshold was 24.2 mÅ (SD 21.7, *P* = 0.02), which corresponded to an increase of 74.7% (SEM 20.4).

##### 3.3.4.2. Electrical pain tolerance

###### 3.3.4.2.1. Intravenous buprenorphine

In the Dahan et al.^5^ RCT, electrical pain tolerance was measured by placing 2 electrodes over the left tibia. The stimulus intensity was increased by 0.5 mA/s, and the maximum cutoff intensity was 128 mA. Subjects were instructed to press a control button when no further increase in stimulus was tolerable. The procedure was performed 3 times, and the mean value represented the electrical pain tolerance at baseline and at the other 13 assessment time points (see Table 1 for exact time points). The baseline pain tolerance in the buprenorphine 0.2 mg and 0.4 mg groups was 16.3 mA (SD 3.9) and 15.0 mA (SD 2.6), respectively. The peak analgesic effect of buprenorphine 0.2 mg occurred at *t* = 75 minutes, which was 6.7 mA (SD 2.8), or 29%, above the baseline value of pain tolerance. The peak analgesic effect of buprenorphine 0.4 mg occurred 130 minutes after administration, which was 23.8 mA (SD 7.4), or 160%, above the baseline value of pain tolerance. Compared with baseline, the peak effect of both doses on pain tolerance was statistically significant.

###### 3.3.4.2.2. Transdermal buprenorphine

In the Koltzenburg et al.^9^ RCT, electrical pain stimuli were delivered to the lateral upper nondominant arm. Eight stimulus trains were delivered every 15 seconds to a maximum intensity of 64 mA. The stimulus intensity was increased in 30% increments until the pain threshold was reached, after which stimulus intensity was increased in 15% increments until pain tolerance was achieved. Compared with baseline, no statistically significant change in pain tolerance was observed at 24 hours and 72 hours following the application of transdermal buprenorphine. Values of pain tolerance at baseline, 24 hours, and 72 hours were not reported.

#### 3.3.5. Primary and secondary hyperalgesia

##### 3.3.5.1. Ultraviolet B light–induced primary and secondary hyperalgesia

###### 3.3.5.1.1. Transdermal buprenorphine

In the Andresen et al.^2^ RCT, ultraviolet B light (290–320 nm) was used to induce first-degree burn injuries on the anterior thigh 24 hours before the assessment of primary and secondary hyperalgesia at baseline and at 24, 72, and 144 hours after application of transdermal buprenorphine. In the published study results,^2^ a discrepancy exists between the assessment time points reported in the methods and results sections. The assessment time points reported in Figure 4^2^ on page 964 are used herein. Mechanical pain and heat pain tolerance were quantified in the area of primary and secondary hyperalgesia using a von Frey filament (size 5.46). The mean baseline values for mechanical pain tolerance and heat pain tolerance were 791.8 kPas^−1^ (SEM 24.8) and 47°C (SEM 0.2), respectively. Within 72 hours, transdermal buprenorphine reduced mechanical pain tolerance in the primary area of hyperalgesia compared with placebo (*P* = 0.006). However, there was no significant effect of buprenorphine on heat pain tolerance in the primary area of hyperalgesia. Fourteen of 20 subjects developed an area of secondary hyperalgesia, but no significant effect of buprenorphine on the area of secondary hyperalgesia was observed for any of the assessed time points.

##### 3.3.5.2. Heat-induced primary and secondary hyperalgesia

###### 3.3.5.2.1. Intravenous buprenorphine

In the Ravn et al.^21^ RCT, a first-degree heat injury was induced using a thermode placed on the lower nondominant leg. Pain associated with primary hyperalgesia was assessed using a visual analog scale at a thermode temperature of 47°C, then 30 seconds later, and every 60 seconds thereafter for a total exposure time of 420 seconds. The area of secondary hyperalgesia was determined using a nylon filament 1, 2, and 3 hours following heat injury. Intravenous buprenorphine 0.3 mg (*P* < 0.0001) and 0.6 mg (*P* < 0.0001) significantly reduced VAS scores associated with primary hyperalgesia compared with placebo. No significant associations were observed between IV buprenorphine 0.3 mg or 0.6 mg and the area of secondary hyperalgesia compared with placebo. All data were presented in bar graphs, and exact values were not reported.

##### 3.3.5.3. Drug-induced secondary hyperalgesia

###### 3.3.5.3.1. Transdermal buprenorphine

In the Andersen et al.^2^ RCT, a 0.1-mL injectate containing 2.5 μg of nerve growth factor was injected into the extensor digitorum longus muscle 10 cm distal to the patella. Following the application of transdermal buprenorphine 20 μg/h, pain tolerance to pressure algometry was assessed at 24, 48, 72, and 144 hours after the injection. The mean baseline pain tolerance was 900.7 kPas^−1^ (SEM 23.3), and no significant effect of nerve growth factor on pressure pain tolerance was observed at any of the assessed time points.

In the Andersen et al.^2^ RCT, a 0.1-mL injectate containing 100 μg of capsaicin was injected at baseline. The injection site was the volar surface of the left forearm, and each injection site was separated by 2 cm. Following the application of transdermal buprenorphine 20 μg/h, secondary hyperalgesia was assessed at 24, 48, 72, and 144 hours after the injection. The area of secondary hyperalgesia was quantified with a von Frey filament (size 5.46), and the area of allodynia was quantified with a soft brush. The mean baseline area of secondary hyperalgesia was 50.8 cm^2^ (SEM 1.6), and the mean baseline area of allodynia was 38.6 cm^2^ (SEM 1.0). No significant effect of capsaicin on the area of secondary hyperalgesia (*P* > 0.9) or allodynia (*P* > 0.7) was observed at any of the assessed time points.

##### 3.3.5.4. Electrically induced secondary mechanical hyperalgesia

###### 3.3.5.4.1. Sublingual and intravenous buprenorphine

In the Koppert et al.^10^ RCT, an area of secondary hyperalgesia was induced by electrically stimulating 2 microdialysis fibers equipped with stainless steel wires inserted intradermally in the central volar region of the forearm. Electrical stimulation was gradually increased during the first 15 minutes of the testing protocol to achieve a pain rating of 5 to 6 on an 11-point numeric rating scale. Thirty minutes after initiation of the electrical stimulation, subjects received either intravenous buprenorphine 0.15 mg during a 5-minute infusion period, sublingual buprenorphine 0.2 mg, or placebo. The area of punctate hyperalgesia was then assessed every 15 minutes using a 450-mN von Frey filament throughout the remaining 150-minute time period of the experimental protocol. Pain ratings obtained >30 minutes after drug or placebo administration decreased significantly in both buprenorphine groups compared with placebo, but no significant differences between the intravenous or sublingual groups were observed. The area of secondary hyperalgesia assessed >30 minutes after drug or placebo administration decreased significantly in both buprenorphine groups compared with placebo, but no significant differences between the intravenous or sublingual groups were observed. The area under the curve (AUC) for the antihyperalgesic and analgesic data was calculated, and the AUC_antihyperalgesia_/AUC_analgesia_ ratio for intravenous and sublingual buprenorphine was 2.6 and 1.9, respectively.

###### 3.3.5.4.2. Intravenous buprenorphine

The Tröster et al.^27^ RCT induced and assessed secondary hyperalgesia using experimental methods similar to those reported in the Koppert et al.^10^ RCT. Thirty minutes after initiation of electrical stimulation, subjects received intravenous buprenorphine 1.5 μg/kg during a 10-minute infusion period or a placebo saline infusion. Pain ratings obtained 65 minutes after buprenorphine administration were reduced 35% (SE 23, *P* < 0.001) compared with baseline. The area of secondary hyperalgesia assessed >30 minutes after buprenorphine administration was reduced 34.4% (SE 32.7, *P* = 0.001) compared with baseline.

#### 3.3.6. Conditioned pain modulation

Two RCTs^3,21^ used the conditioned pain modulation (CPM) test. In general, CPM compares pain induced by a test stimulus with pain induced by an identical test stimulus after exposure to a conditioning stimulus, which is typically the cold pressor test. One RCT used intravenous buprenorphine^21^ and 1 used transdermal buprenorphine.^3^

##### 3.3.6.1. Heat as the test stimuli

###### 3.3.6.1.1. Intravenous buprenorphine

In the Ravn et al.^21^ RCT, the test stimulus was a repeated series of heat stimuli at 47°C applied to the nondominant lower leg for 4 seconds. Twelve seconds later, the nondominant hand was submerged in a cold bath (0.3°C–0.5°C) for 30 seconds (the conditioning stimulus). At the approximate mid-point of the cold bath submersion and then 12 seconds and 24 seconds after removal of the hand from the cold bath, the test stimulus was applied. During application of the test stimulus, pain intensity was assessed using a VAS. The CPM efficiency was calculated by summing the VAS scores from the second, third, and fourth test stimuli divided by the VAS score from the first test stimuli multiplied by 3. The quotient of this equation was then subtracted from 1 and multiplied by 100 to yield the percentage of CPM efficiency. Using this approach to quantify CPM efficiency, buprenorphine 0.6 mg but not 0.3 mg was associated with a significant analgesic effect compared with placebo (*P* = 0.004).

###### 3.3.6.1.2. Transdermal buprenorphine

In the Arndt-Nielsen et al.^3^ RCT, CPM measurements were obtained before placement of transdermal buprenorphine and then at 24, 48, and 72 hours after transdermal application. The test stimulus was pressure pain tolerance assessed using a handheld algometer with an increase rate of 30 kPa/s. The test stimulus was applied to the left arm 10 cm distal to the elbow on the lateral aspect of the extensor digitor muscle. The conditioning stimulus was the cold pressor test performed by submerging the right hand in a circulating cold bath (3.0°C ± 0.3°C) for 2 minutes. Conditioned pain modulation was performed by applying the test stimulus before the conditioning stimulus and then reapplying the test stimulus immediately after the conditioning stimulus and again at 2 and 5 minutes after the conditioning stimulus. The percent increase in the test stimulus was significantly elevated (*P* = 0.004) immediately after completion of the conditioning stimulus “during the 72-hour period”^3^ compared with placebo. The data were presented graphically, and exact values were not reported. In addition, statistical values for the percent change in the test stimulus for each assessment period at 24, 48, and 72 hours compared with placebo were not reported.

#### 3.3.7. Nociceptive flexion reflex

One RCT^6^ assessed the effects of buprenorphine on the nociceptive flexion reflex (NFR) test. The NFR is a polysynaptic reflex that, when activated by pain stimuli, produces withdrawal of the targeted body region.^24^

##### 3.3.7.1. Electrical threshold

###### 3.3.7.1.1. Intravenous buprenorphine

In the Escher et al.^6^ RCT, the sural nerve was stimulated in the area of the retromalleolar track using surface electrodes. The stimulus was a single rectangular impulse (0.5 ms) delivered with a 6- to 10-second interstimulus interval using a constant current stimulator. The electromyographic responses were recorded using surface electrodes placed over the tendon of the ipsilateral biceps femoris. The threshold for the NFR was defined as a multiphasic signal recorded within 90 milliseconds to <250 milliseconds and occurring in a corrected computed surface area of >0.5 mV/ms. The mean baseline NFR threshold was 31.6 mÅ (SD 9.5), and intravenous buprenorphine significantly increased the threshold for >4 hours. The maximum NFR increase was 14.1 mÅ (SD 17.5, *P* = 0.02), which corresponded to an increase of 53.7% (SEM 20.2).

## 4. Discussion

The main findings of this meta-narrative systematic review suggest that the effects of buprenorphine on pain perception in healthy humans are influenced by the route of buprenorphine administration, buprenorphine dose, and pain stimulus modality. In addition, considerable differences exist in the effects of intravenous and transdermal buprenorphine on pain perception, but only 1 study used sublingual buprenorphine, which limited comparison with the other drug formulations. The study findings are summarized in Table 3.

**Table 3:** Summary effects of buprenorphine on pain perception in healthy adults.

Stimulus modality	Buprenorphine route of administration		
	Intravenous	Transdermal	Sublingual
Heat pain			
Threshold	0	+	—
Tolerance	—	+	—
Cold pressor			
Threshold	+*†	—	—
Tolerance	0*/+†	0	—
Cool detection			
Threshold	+	—	—
Tolerance	—	—	—
Mechanical			
Threshold	—	—	—
Tolerance	0*/+†	+	—
Electrical			
Threshold	+	0	—
Tolerance	+*†	0	—
Hyperalgesia/allodynia			
Primary hyperalgesia	+‡	0§‖/+¶	—
Secondary hyperalgesia	0#/+**††	0§¶**	+#††
Condition pain modulation	0*/+†	+	—
Nociceptive flexion reflex	+	—	—

0, no change; (+), increased; —, no data.

* Lower dose.

† Higher dose.

‡ Heat pain perception to heat-induced hyperalgesia.

§ Heat pain tolerance to light-induced hyperalgesia.

‖ Pressure algometry response to nerve growth factor–induced primary hyperalgesia.

¶ Mechanical pain tolerance to ultraviolet light–induced hyperalgesia.

# Mechanical pain to heat-induced secondary hyperalgesia.

** Mechanical pain response to capsaicin-induced secondary hyperalgesia and allodynia.

†† Reduction in area of electrically induced secondary hyperalgesia.

Exposure to heat activates small unmyelinated C-fibers that have a mean diameter of 1 μm and velocities ranging from 0.2 to 2 m/s.^16,17^ Intravenous buprenorphine administered as a single dose had no effect on heat pain threshold, but transdermal buprenorphine at 24 hours and 72 hours increased both heat pain threshold and tolerance. The effects of transdermal buprenorphine on heat pain perception are consistent with the reported pharmacokinetics of transdermal administration, where the minimal effective therapeutic dose of a 35-μg patch was achieved at 21 (SD 16) hours following application in healthy adults, and peak plasma concentrations were reached at 60 hours.^11^ These observations suggest that (1) longer periods of buprenorphine administration at peak serum levels may be necessary to significantly increase heat pain thresholds and tolerances and (2) measures of heat pain perception may not be significantly affected by single intravenous doses of buprenorphine.

Exposure to cold and coolness stimuli activates myelinated A-delta fibers that have mean diameters <3 μm and conduction velocities ranging from 12 to 30 m/s.^17^ Higher and lower doses of intravenous buprenorphine increased cold pressor pain and cool detection thresholds, but the effects on cold pressor tolerance were mixed. At lower doses, intravenous buprenorphine had no effect on cold pressor pain tolerance, but higher doses were associated with significant elevations in pain tolerance. Transdermal buprenorphine at 20 μg/h and 35 μg/h was associated with significant elevations in cold pressor pain thresholds. These observations suggest that (1) cold pressor pain and cool detection thresholds are responsive to both lower and higher doses of intravenous buprenorphine, (2) cold pressor pain tolerance may only be responsive to higher doses of intravenous buprenorphine, and (3) cold pressor pain tolerance is responsive to transdermal buprenorphine.

Mechanical and electrical stimuli activate myelinated A-beta fibers that have a mean diameter of 8 μm and conduction velocities ranging from 30 to 70 m/s.^17^ Intravenous buprenorphine significantly increased electrical pain thresholds and tolerances, but transdermal buprenorphine had no significant effects. Higher, but not lower, doses of intravenous buprenorphine and transdermal buprenorphine increased mechanical pain tolerance. These findings suggest that (1) electrical pain thresholds and tolerances are responsive to intravenous buprenorphine and (2) mechanical pain tolerance is responsive to transdermal buprenorphine and higher doses of intravenous buprenorphine.

Primary and secondary hyperalgesia activate unmyelinated C-fibers and myelinated A-delta fibers.^4^ Intravenous, sublingual, and transdermal buprenorphine attenuated primary and secondary hyperalgesia. The conditioned pain modulation test activates a broad range of neural fibers, including unmyelinated C-fibers, myelinated A-delta fibers, and the descending inhibitory spinal pathways originating in the periaqueductal gray and rostral ventromedial medulla regions.^18,28^ Higher doses of intravenous buprenorphine and transdermal buprenorphine were associated with improved conditioned pain modulation efficiency. The nociceptive flexion reflex is elicited by the activation of myelinated A-delta fibers,^24^ and intravenous buprenorphine significantly increased the threshold for eliciting the reflex.

The observations from this study should be carefully interpreted with knowledge of the clinical heterogeneity identified in this systematic review. First, the methods used to calculate buprenorphine doses varied between studies, which limited quantifying an accurate value for low and high doses of intravenous buprenorphine. Second, the infusion time of intravenous buprenorphine ranged from 1 minute to 210 minutes. Thus, it was not possible to quantify the optimal duration of the intravenous buprenorphine infusion. Third, variations in the methods for conducting the experimental tests of pain perception were identified, which could impact the reported summary effects of buprenorphine on pain perception. Fourth, these findings do not provide evidence supporting or refuting the potential analgesic effects of buprenorphine in real-world clinical practice.

Based on the summary findings of this meta-narrative systematic review, considerations for clinical trials involving healthy subjects are proposed (Table 4). Although these suggestions should be cautiously interpreted due to clinical heterogeneity, this body of evidence provides a structured framework for advancing the use of experimental tests of pain perception in clinical trials of buprenorphine involving comparator groups of healthy adults.

**Table 4:** Considerations for incorporating experimental tests of pain perception for healthy subjects in future clinical trials of buprenorphine.

Buprenorphine	Stimulus modality
Intravenous	Cold pressor Pain threshold Pain tolerance (higher doses)
	Mechanical Pain tolerance
	Electrical Pain threshold Pain tolerance (lower and higher doses)
	Hyperalgesia Primary hyperalgesia Secondary hyperalgesia (stimulus dependent)
	Conditioned pain modulation Higher doses
	Nociceptive flexion reflex
Sublingual	Hyperalgesia Secondary hyperalgesia
Transdermal	Hyperalgesia Primary hyperalgesia (stimulus dependent) Secondary hyperalgesia

## Disclosures

The authors have no conflicts of interest to report.

## Appendix A. Supplemental digital content

Supplemental digital content associated with this article can be found online at http://links.lww.com/PR9/A317.

## Supplementary Material

SUPPLEMENTARY MATERIAL

## References

[1] Aiyer R Gulati A Gungor S Bhatia A Mehta N. Treatment of chronic pain with various buprenorphine formulations: a systematic review of clinical studies. Anesth Analg 2018;127:529–38. 29239947 10.1213/ANE.0000000000002718

[2] Andresen T Staahl C Oksche A Mansikka H Arendt-Nielsen L Drewes AM. Effect of transdermal opioids in experimentally induced superficial, deep and hyperalgesic pain. Br J Pharmacol 2011;164:934–45. 21182491 10.1111/j.1476-5381.2010.01180.x PMC3195916

[3] Arendt-Nielsen L Andresen T Malver LP Oksche A Mansikka H Drewes AM. A double-blind, placebo-controlled study on the effect of buprenorphine and fentanyl on descending pain modulation: a human experimental study. Clin J Pain 2012;28:623–7. 22156892 10.1097/AJP.0b013e31823e15cb

[4] Coutaux A Adam F Willer JC Le Bars D. Hyperalgesia and allodynia: peripheral mechanisms. Joint Bone Spine 2005;72:359–71. 16214069 10.1016/j.jbspin.2004.01.010

[5] Dahan A Yassen A Romberg R Sarton E Teppema L Olofsen E Danhof M. Buprenorphine induces ceiling in respiratory depression but not in analgesia. Br J Anaesth 2006;96:627–32. 16547090 10.1093/bja/ael051

[6] Escher M Daali Y Chabert J Hopfgartner G Dayer P Desmeules J. Pharmacokinetic and pharmacodynamic properties of buprenorphine after a single intravenous administration in healthy volunteers: a randomized, double-blind, placebo-controlled, crossover study. Clin Ther 2007;29:1620–31. 17919544 10.1016/j.clinthera.2007.08.007

[7] Greenhalgh T Robert G Macfarlane F Bate P Kyriakidou O Peacock R. Storylines of research in diffusion of innovation: a meta-narrative approach to systematic review. Soc Sci Med 2005;61:417–30. 15893056 10.1016/j.socscimed.2004.12.001

[8] Hay JL La Vincente SF Somogyi AA Chapleo CB White JM. Potentiation of buprenorphine antinociception with ultra-low dose naltrexone in healthy subjects. Eur J Pain 2011;15:293–8. 20728384 10.1016/j.ejpain.2010.07.009

[9] Koltzenburg M Pokorny R Gasser UE Richarz U. Differential sensitivity of three experimental pain models in detecting the analgesic effects of transdermal fentanyl and buprenorphine. PAIN 2006;126:165–74. 16901645 10.1016/j.pain.2006.06.028

[10] Koppert W Ihmsen H Körber N Wehrfritz A Sittl R Schmelz M Schüttler J. Different profiles of buprenorphine-induced analgesia and antihyperalgesia in a human pain model. PAIN 2005;118:15–22. 16154698 10.1016/j.pain.2005.06.030

[11] Kress HG. Clinical update on the pharmacology, efficacy and safety of transdermal buprenorphine. Eur J Pain 2009;13:219–30. 18567516 10.1016/j.ejpain.2008.04.011

[12] La Vincente SF White JM Somogyi AA Bochner F Chapleo CB. Enhanced buprenorphine analgesia with the addition of ultra-low-dose naloxone in healthy subjects. Clin Pharmacol Ther 2008;83:144–52. 17568402 10.1038/sj.clpt.6100262

[13] Lutfy K Cowan A. Buprenorphine: a unique drug with complex pharmacology. Curr Neuropharmacol 2004;2:395–402. 18997874 10.2174/1570159043359477 PMC2581407

[14] Mammen K Bell J. The clinical efficacy and abuse potential of combination buprenorphine-naloxone in the treatment of opioid dependence. Expert Opin Pharmacother 2009;10:2537–44. 19708849 10.1517/14656560903213405

[15] Moher D Liberati A Tetzlaff J Altman DG, PRISMA Group. Preferred reporting items for systematic reviews and meta-analyses: the PRISMA statement. BMJ 2009;339:b2535. 19622551 10.1136/bmj.b2535 PMC2714657

[16] Nordin M. Low-threshold mechanoreceptive and nociceptive units with unmyelinated (C) fibres in the human supraorbital nerve. J Physiol 1990;426:229–40. 2231398 10.1113/jphysiol.1990.sp018135 PMC1189885

[17] Olausson H Marshall A Nagi SS Cole J. Slow touch and ultrafast pain fibres: revisiting peripheral nerve classification. Clin Neurophysiol 2024;163:255–62. 38704307 10.1016/j.clinph.2024.04.008

[18] Ossipov MH Dussor GO Porreca F. Central modulation of pain. J Clin Invest 2010;120:3779–87. 21041960 10.1172/JCI43766 PMC2964993

[19] Pergolizzi J Aloisi AM Dahan A Filitz J Langford R Likar R Mercadante S Morlion B Raffa RB Sabatowski R Sacerdote P Torres LM Weinbroum AA. Current knowledge of buprenorphine and its unique pharmacological profile. Pain Pract 2010;10:428–50. 20492579 10.1111/j.1533-2500.2010.00378.x

[20] Raffa RB Haidery M Huang HM Kalladeen K Lockstein DE Ono H Shope MJ Sowunmi OA Tran JK Pergolizzi JV Jr. The clinical analgesic efficacy of buprenorphine. J Clin Pharm Ther 2014;39:577–83. 25070601 10.1111/jcpt.12196

[21] Ravn P Secher EL Skram U Therkildsen T Christrup LL Werner MU. Morphine- and buprenorphine-induced analgesia and antihyperalgesia in a human inflammatory pain model: a double-blind, randomized, placebo-controlled, five-arm crossover study. J Pain Res 2013;6:23–38. 23359655 10.2147/JPR.S36827 PMC3555550

[22] Sadée W Rosenbaum JS Herz A. Buprenorphine: differential interaction with opiate receptor subtypes in vivo. J Pharmacol Exp Ther 1982;223:157–62. 6288917

[23] Singhal A Tripathi BM Pal HR Jena R Jain R. Subjective effects of additional doses of buprenorphine in patients on buprenorphine maintenance. Addict Behav 2007;32:320–31. 16814937 10.1016/j.addbeh.2006.05.003

[24] Skljarevski V Ramadan NM. The nociceptive flexion reflex in humans—review article. PAIN 2002;96:3–8. 11932055 10.1016/s0304-3959(02)00018-0

[25] Sterne JAC Savović J Page MJ Elbers RG Blencowe NS Boutron I Cates CJ Cheng HY Corbett MS Eldridge SM Emberson JR Hernán MA Hopewell S Hróbjartsson A Junqueira DR Jüni P Kirkham JJ Lasserson T Li T McAleenan A Reeves BC Shepperd S Shrier I Stewart LA Tilling K White IR Whiting PF Higgins JPT. RoB 2: a revised tool for assessing risk of bias in randomised trials. BMJ 2019;366:l4898. 31462531 10.1136/bmj.l4898

[26] Tejwani GA Rattan AK. The role of spinal opioid receptors in antinociceptive effects produced by intrathecal administration of hydromorphone and buprenorphine in the rat. Anesth Analg 2002;94:1542–6. Table of contents. 12032023 10.1097/00000539-200206000-00031

[27] Tröster A Ihmsen H Singler B Filitz J Koppert W. Interaction of fentanyl and buprenorphine in an experimental model of pain and central sensitization in human volunteers. Clin J Pain 2012;28:705–11. 22469638 10.1097/AJP.0b013e318241d948

[28] Villanueva L Le Bars D. The activation of bulbo-spinal controls by peripheral nociceptive inputs: diffuse noxious inhibitory controls. Biol Res 1995;28:113–25. 8728826

[29] Webster L Gudin J Raffa RB Kuchera J Rauck R Fudin J Adler J Mallick-Searle T. Understanding buprenorphine for use in chronic pain: expert opinion. Pain Med 2020;21:714–23. 31917418 10.1093/pm/pnz356 PMC7139205

[30] Weimer MB Morford KL. Buprenorphine for opioid use Disorder-An essential medical treatment. JAMA Int Med 2024;184:1248–9. 10.1001/jamainternmed.2024.3977 39186298

[31] Wong G Greenhalgh T Westhorp G Pawson R. Development of methodological guidance, publication standards and training materials for realist and meta-narrative reviews: The RAMESES (realist and meta-narrative evidence syntheses—evolving standards) project. Southampton, UK: National Institute of Health Research Journals Research; 2014. 25642521

[32] Zheng D Moman R Hooten WM. Effect of buprenorphine and naltrexone on hyperalgesia: a systematic review. York: National Institute for Health and Care Research; https://www.crd.york.ac.uk/prospero/display_record.php?ID=CRD42020134850 (2024, Accessed September 15, 2024).

