# Supplementary material for: Effects of buprenorphine on pain perception in healthy adults: a meta-narrative systematic review
Source: Pain Rep. 2025 May 20;10(3):e1294. doi: 10.1097/PR9.0000000000001294 (PMC12094366; doi:10.1097/PR9.0000000000001294)
Supplement: SUPPLEMENTARY MATERIAL [file painreports-10-e1294-s001.pdf]

Ovid

Databases: APA PsycInfo 1806 to July Week 2 2024, EBM Reviews - Cochrane Central Register of Controlled Trials June 2024, EBM Reviews - Cochrane Database of Systematic Reviews 2005 to July 10, 2024, Embase 1974 to 2024 July 15, Ovid MEDLINE(R) and Epub Ahead of Print, In-Process, In-Data-Review & Other Non-Indexed Citations, Daily and Versions 1946 to July 15, 2024

Search Strategy:

#	Searches	Results
1	exp Pain Perception/	117793
2	exp Hyperalgesia/	43698
3	(((electrical or mechanical* or thermal* or heat or hot or temperature or cold or pressure* or tactile or touch or "opioid induced") adj4 (sensitiv* or hypersensitiv* or sensation*)) or (pain* adj4 (sensitiv* or hypersensitiv* or sensation* or perception* or persisten* or threshold* or tolerance* or tolerat* or sense)) or allodyni* or antianalges* or "anti-analges*" or antinocicept* or "anti-nocicept*" or hyperalg* or hyperalgesi* or hyperesthesi* or hyperpathi* or nocicept* or nocipercept*).ti,ab,hw,kw.	539409
4	1 or 2 or 3	543063
5	exp Buprenorphine/	40152
6	("6029 m" or 6029m or "6029-m" or buprenex or buprenorphine or buprex or prefin or "rx 6029 m" or rx6029m or "rx-6029-m" or subutex or temgesic).ti,ab,hw,kw.	48852
7	5 or 6	48852
8	exp Naltrexone/	30576
9	(antaxone or celupan or "en 1639a" or en1639a or "en-1639a" or nalorex or naltrexone or nemexin or revia or trexan).ti,ab,hw,kw.	38231
10	8 or 9	38231
11	7 or 10	82099
12	4 and 11	6045
13	exp meta analysis/	533016
14	exp Meta-Analysis as Topic/	88225
15	exp "systematic review"/	743008
16	exp controlled study/	10688200
17	exp Randomized Controlled Trial/	1454129
18	exp triple blind procedure/	541
19	exp Double-Blind Method/	571973
20	exp Single-Blind Method/	116680
21	exp latin square design/	455
22	exp Placebos/	481938

23	exp Placebo Effect/	15875
24	exp comparative study/	3663205
25	exp intervention studies/	68666
26	exp Cross-Sectional Studies/	1167073
27	exp Cross-Over Studies/	184032
28	exp Cohort Studies/	4029632
29	exp longitudinal study/	399836
30	exp retrospective study/	2884479
31	exp prospective study/	1748301
32	exp population research/	141074
33	exp observational study/	540886
34	exp clinical trial/	2935393
35	clinical study/	173417
36	exp case-control studies/	1789253
37	exp confidence interval/	198024
38	exp multivariate analysis/	744830
39	((evidence adj based) or (meta adj analys*) or metaanalys* or (systematic* adj3 review*) or guideline* or (control* adj3 study) or (control* adj3 trial) or (randomized adj3 study) or (randomized adj3 trial) or (randomised adj3 study) or (randomised adj3 trial) or "pragmatic clinical trial" or (random* adj1 allocat*) or (doubl* adj blind*) or (doubl* adj mask*) or (singl* adj blind*) or (singl* adj mask*) or (tripl* adj blind*) or (tripl* adj mask*) or (trebl* adj blind*) or (trebl* adj mask*) or "latin square" or placebo* or nocebo* or multivariate or "comparative study" or "comparative survey" or "comparative analysis" or (intervention* adj2 study) or (intervention* adj2 trial) or "cross-sectional study" or "cross-sectional analysis" or "cross-sectional survey" or "cross-sectional design" or "prevalence study" or "prevalence analysis" or "prevalence survey" or "disease frequency study" or "disease frequency analysis" or "disease frequency survey" or crossover or "cross-over" or cohort* or "longitudinal study" or "longitudinal survey" or "longitudinal analysis" or "longitudinal evaluation" or longitudinal* or ((retrospective or "ex post facto") adj3 (study or survey or analysis or design)) or retrospectiv* or "prospective study" or "prospective survey" or "prospective analysis" or prospectiv* or "concurrent study" or "concurrent survey" or "concurrent analysis" or "clinical study" or "clinical trial" or (("phase 0" or "phase 1" or "phase I" or "phase 2" or "phase II" or "phase 3" or "phase III" or "phase 4" or "phase IV") adj5 (trial or study)) or "case control study" or "case base study" or "case referrent study" or "case referent study" or "case referent study" or "case compeer study" or "case comparison study" or "matched case control" or "multicenter study" or "multi-center study" or "odds ratio" or "confidence interval" or "change analysis" or ((study or trial or random* or control*) and compar*) or (parallel adj4 (trial or study))).mp,pt.	33042877
40	or/13-39	33706007

41	12 and 40	3381
42	limit 41 to ("all adult (19 plus years)" or "young adult (19 to 24 years)" or "adult (19 to 44 years)" or "young adult and adult (19-24 and 19-44)" or "middle age (45 to 64 years)" or "middle aged (45 plus years)" or "all aged (65 and over)" or "aged (80 and over)") [Limit not valid in APA PsycInfo,CCTR,CDSR,Embase; records were retained]	2786
43	limit 42 to (adult <18 to 64 years> or aged <65+ years>) [Limit not valid in APA PsycInfo,CCTR,CDSR,Ovid MEDLINE(R); records were retained]	899
44	limit 41 to ("all infant (birth to 23 months)" or "all child (0 to 18 years)" or "newborn infant (birth to 1 month)" or "infant (1 to 23 months)" or "preschool child (2 to 5 years)" or "child (6 to 12 years)" or "adolescent (13 to 18 years)") [Limit not valid in APA PsycInfo,CCTR,CDSR,Embase; records were retained]	2687
45	limit 44 to (embryo or infant or child or preschool child <1 to 6 years> or school child <7 to 12 years> or adolescent <13 to 17 years>) [Limit not valid in APA PsycInfo,CCTR,CDSR,Ovid MEDLINE(R); records were retained]	255
46	45 not 43	33
47	41 not 46	3348
48	(exp animals/ or exp nonhuman/) not exp humans/	12615898
49	((alpaca or alpacas or amphibian or amphibians or animal or animals or antelope or armadillo or armadillos or avian or baboon or baboons or beagle or beagles or bee or bees or bird or birds or bison or bovine or buffalo or buffaloes or buffalos or "c elegans" or "Caenorhabditis elegans" or camel or camels or canine or canines or carp or cats or cattle or chick or chicken or chickens or chicks or chimp or chimpanze or chimpanzees or chimps or cow or cows or "D melanogaster" or "dairy calf" or "dairy calves" or deer or dog or dogs or donkey or donkeys or drosophila or "Drosophila melanogaster" or duck or duckling or ducklings or ducks or equid or equids or equine or equines or feline or felines or ferret or ferrets or finch or finches or fish or flatworm or flatworms or fox or foxes or frog or frogs or "fruit flies" or "fruit fly" or "G mellonella" or "Galleria mellonella" or geese or gerbil or gerbils or goat or goats or goose or gorilla or gorillas or hamster or hamsters or hare or hares or heifer or heifers or horse or horses or insect or insects or jellyfish or kangaroo or kangaroos or kitten or kittens or lagomorph or lagomorphs or lamb or lambs or llama or llamas or macaque or macaques or macaw or macaws or marmoset or marmosets or mice or minipig or minipigs or mink or minks or monkey or monkeys or mouse or mule or mules or nematode or nematodes or octopus or octopuses or orangutan or "orang-utan" or orangutans or "orang-utans" or oxen or parrot or parrots or pig or pigeon or pigeons or piglet or piglets or pigs or porcine or primate or primates or quail or rabbit or rabbits or rat or rats or reptile or reptiles or rodent or rodents or ruminant or ruminants or salmon or sheep or shrimp or slug or slugs or swine or tamarin or tamarins or toad or toads or trout or urchin or urchins or vole or voles or waxworm or waxworms or worm or worms or xenopus or "zebra fish" or zebrafish) not (human or humans or patient or patients)).ti,ab,hw,kw.	11147467
50	47 not (48 or 49)	1588

51	limit 50 to (editorial or erratum or note or addresses or autobiography or bibliography or biography or blogs or comment or dictionary or directory or interactive tutorial or interview or lectures or legal cases or legislation or news or newspaper article or overall or patient education handout or periodical index or portraits or published erratum or video-audio media or webcasts) [Limit not valid in APA PsycInfo,CCTR,CDSR,Embase,Ovid MEDLINE(R); records were retained]	31
52	from 51 keep 4-6	3
53	(50 not 51) or 52	1560
54	remove duplicates from 53	1209

Scopus

1 TITLE-ABS-KEY(((electrical or mechanical* or thermal* or heat or hot or temperature or cold or pressure* or tactile or touch or "opioid induced") W/4 (sensitiv* or hypersensitiv* or sensation*)) or (pain* W/4 (sensitiv* or hypersensitiv* or sensation* or perception* or persisten* or threshold* or tolerance* or tolerat* or sense)) or allodyni* or antianalges* or "anti-analges*" or antinocicept* or "anti-nocicept*" or hyperalg* or hyperalgesi* or hyperesthesi* or hyperpathi* or nocicept* or nocipercept*)

2 TITLE-ABS-KEY("6029 m" or 6029m or "6029-m" or buprenex or buprenorphine or buprex or prefin or "rx 6029 m" or rx6029m or "rx-6029-m" or subutex or temgesic)

3 TITLE-ABS-KEY(antaxone OR celupan OR "en 1639a" OR en1639a OR "en-1639a" OR nalorex OR naltrexone OR nemexin OR revia OR trexan)

4 TITLE-ABS-KEY((evidence W/1 based) OR (meta W/1 analys*) OR metaanalys* OR (systematic* W/3 review*) OR guideline* OR (control* W/3 study) OR (control* W/3 trial) OR (randomized W/3 study) OR (randomized W/3 trial) OR (randomised W/3 study) OR (randomised W/3 trial) OR "pragmatic clinical trial" OR (random* W/1 allocat*) OR (doubl* W/1 blind*) OR (doubl* W/1 mask*) OR (singl* W/1 blind*) OR (singl* W/1 mask*) OR (tripl* W/1 blind*) OR (tripl* W/1 mask*) OR (trebl* W/1 blind*) OR (trebl* W/1 mask*) OR "latin square" OR placebo* OR nocebo* OR multivariate OR "comparative study" OR "comparative survey" OR "comparative analysis" OR (intervention* W/2 study) OR (intervention* W/2 trial) OR "cross-sectional study" OR "cross-sectional analysis" OR "cross-sectional survey" OR "cross-sectional design" OR "prevalence study" OR "prevalence analysis" OR "prevalence survey" OR "disease frequency study" OR "disease frequency analysis" OR "disease frequency survey" OR crossover OR "cross-over" OR cohort* OR "longitudinal study" OR "longitudinal survey" OR "longitudinal analysis" OR "longitudinal evaluation" OR longitudinal* OR ((retrospective OR "ex post facto") W/3 (study OR survey OR analysis OR design)) OR retrospectiv* OR "prospective study" OR "prospective survey" OR "prospective analysis" OR prospectiv* OR "concurrent study" OR "concurrent survey" OR "concurrent analysis" OR "clinical study" OR "clinical trial" OR (("phase 0" or "phase 1" or "phase I" or "phase 2" or "phase II" or "phase 3" or "phase III" or "phase 4" or "phase IV") W/5 (trial or study)) OR "case control study" OR "case base study" OR "case referrent study" OR "case referent study" OR "case referent study" OR "case compeer study" OR "case comparison study" OR "matched case control" OR "multicenter study" OR "multi-center study" OR "odds ratio" OR "confidence interval" OR "change analysis" OR ((study OR trial OR random* OR control*) AND compar*) OR (parallel W/4 (trial or study)))

5 1 and (2 or 3) and 4

6 TITLE-ABS-KEY(newborn* or neonat* or infant* or toddler* or child* or adolescent* or paediatric* or pediatric* or girl or girls or boy or boys or teen or teens or teenager* or preschooler* or "pre-schooler*" or preteen or preteens or "pre-teen" or "pre-teens" or youth or youths) AND NOT TITLE-ABS-KEY(adult or adults or "middle age" or "middle aged" OR elderly OR geriatric* OR "old people" OR "old person*" OR "older people" OR "older person*" OR "very old")

7 5 and not 6

8 TITLE-ABS-KEY((alpaca OR alpacas OR amphibian OR amphibians OR animal OR animals OR antelope OR armadillo OR armadillos OR avian OR baboon OR baboons OR

beagle OR beagles OR bee OR bees OR bird OR birds OR bison OR bovine OR buffalo OR buffaloes OR buffalos OR "c elegans" OR "Caenorhabditis elegans" OR camel OR camels OR canine OR canines OR carp OR cats OR cattle OR chick OR chicken OR chickens OR chicks OR chimp OR chimpanze OR chimpanzees OR chimps OR cow OR cows OR "D melanogaster" OR "dairy calf" OR "dairy calves" OR deer OR dog OR dogs OR donkey OR donkeys OR drosophila OR "Drosophila melanogaster" OR duck OR duckling OR ducklings OR ducks OR equid OR equids OR equine OR equines OR feline OR felines OR ferret OR ferrets OR finch OR finches OR fish OR flatworm OR flatworms OR fox OR foxes OR frog OR frogs OR "fruit flies" OR "fruit fly" OR "G mellonella" OR "Galleria mellonella" OR geese OR gerbil OR gerbils OR goat OR goats OR goose OR gorilla OR gorillas OR hamster OR hamsters OR hare OR hares OR heifer OR heifers OR horse OR horses OR insect OR insects OR jellyfish OR kangaroo OR kangaroos OR kitten OR kittens OR lagomorph OR lagomorphs OR lamb OR lambs OR llama OR llamas OR macaque OR macaques OR macaw OR macaws OR marmoset OR marmosets OR mice OR minipig OR minipigs OR mink OR minks OR monkey OR monkeys OR mouse OR mule OR mules OR nematode OR nematodes OR octopus OR octopuses OR orangutan OR "orang-utan" OR orangutans OR "orang-utans" OR oxen OR parrot OR parrots OR pig OR pigeon OR pigeons OR piglet OR piglets OR pigs OR porcine OR primate OR primates OR quail OR rabbit OR rabbits OR rat OR rats OR reptile OR reptiles OR rodent OR rodents OR ruminant OR ruminants OR salmon OR sheep OR shrimp OR slug OR slugs OR swine OR tamarin OR tamarins OR toad OR toads OR trout OR urchin OR urchins OR vole OR voles OR waxworm OR waxworms OR worm OR worms OR xenopus OR "zebra fish" OR zebrafish) AND NOT (human OR humans or patient or patients))

9 7 and not 8

10 DOCTYPE(ed) OR DOCTYPE(bk) OR DOCTYPE(er) OR DOCTYPE(no) OR DOCTYPE(sh)

11 9 and not 10

12 INDEX(embase) OR INDEX(medline) OR PMID(0* OR 1* OR 2* OR 3* OR 4* OR 5* OR 6* OR 7* OR 8* OR 9*)

13 11 and not 12
